# Editorial: Emerging trends in the quality check of herbal medicines, supplements, and “botanicals”

**DOI:** 10.3389/fphar.2025.1715714

**Published:** 2025-10-30

**Authors:** Niccolò Pilla, Daniel Dias Rufino Arcanjo, Massimo Lucarini, Alessandra Durazzo

**Affiliations:** ^1^ Università di Torino, Turin, Italy; ^2^ Università Campus Bio-Medico di Roma, Rome, Italy; ^3^ LAFMOL–Laboratory of Functional and Molecular Studies in Physiopharmacology, Department of Biophysics and Physiology, Federal University of Piauí, Teresina, Piauí, Brazil; ^4^ CREA-Research Centre for Food and Nutrition, Rome, Italy

**Keywords:** botanicals, quality assessment and control, emerging technologies, metrology, data management

## 1 Introduction

This Research Topic aims to explore and discuss emerging trends in the quality assessment of herbal medicines, supplements, and botanicals.

To gather further insights in the quality assessment of herbal products the following topics were addressed: comparative analysis of chemical and spectrophotometric techniques for quality assessment; identification and validation of quality markers in herbal products; development of sustainable and innovative analytical methods; challenges and solutions in standardizing quality checks for diverse herbal matrices; and management and utilization of data and databases in herbal medicine research. In this context, the present Research Topic, Emerging Trends in the Quality Check of Herbal Medicines, Supplements, and “Botanicals”, brings together 10 contributions that address these matters well from different points of view.

Some authors presented the development of new methodologies or applications and case studies of research. For instance, Wang et al. presented a quality evaluation of *Polygonatum sibiricum* slices from different regions based on appearance traits and multi-index metabolites combined with a technique for order preference by similarity to ideal solution—TOPSIS—and gray relation analysis. Zhou et al. aimed to authenticate Renshen Jianpi Wan (RSJPW), a classical CCPP composed of 11 prescribed botanical drugs, using DNA metabarcoding to overcome challenges in species-level identification of processed biological ingredients.

Xin et al. investigated metabolomic and lipidomic profiling of traditional Chinese medicine *Testudinis Carapax et Plastrum* and its substitutes.

Luis and Schneider reported a large variability in the alkaloid content of *Corydalis yanhusuo* dietary supplements available online in the United States.

Xu et al. studied the efficacy of Hongjing I granule, an herbal medicine, in patients with mild to moderate erectile dysfunction in a randomized controlled trial.

Bai and Dong presented the development and validation of the HPLC–MS/MS method and its application in the pharmacokinetic study of the Mongolian drug Sendeng-4 in rat blood plasma.

Lenssen and de Boer studied, through an analysis of EU and national case law from the Netherlands, including self-regulatory decision-making, the implications of case law on botanical health claims.

It is worth mentioning the article by Harnly et al. that carried out a review focused on one-class modeling for verification of botanical identity.

Hao et al. presented a review of botany, application, processing, phytochemistry, quality control, pharmacology, and toxicology of *Euodiae Fructus*.

Al-Sammarraie et al., by carrying out an artificial intelligence-aided scoping review of medicinal plant research in the Fertile Crescent, showed that the number of ethnobotanical studies was limited, suggesting an urgent need to prevent the loss of ancestral knowledge by formalizing it through evidence-based research and policy guidelines. Al-Sammarraie et al. suggested that to address these gaps through interdisciplinary collaboration and improved data-sharing, mechanisms will be crucial for advancing Traditional Arabic and Islamic Medicine research and medicinal plants.

